# A negative feedback loop between XBP1 and Fbw7 regulates cancer development

**DOI:** 10.1038/s41389-019-0124-4

**Published:** 2019-02-19

**Authors:** Unbin Chae, Heejin Lee, Bokyung Kim, Haiyoung Jung, Byeong Mo Kim, Ann- Hwee Lee, Dong-Seok Lee, Sang-Hyun Min

**Affiliations:** 1 School of Life Sciences and Biotechnology, BK21 Plus KNU Creative BioResearch Group, Kyungpook National University, Daegu, Republic of Korea; 2 Drug Development Center, DGMIF, Daegu, Republic of Korea; 3 Department of Neurology, School of Medicine, UC Davis, Davis, CA 95817 USA; 4 Immunotherapy Convergence Research Center, Korea Research Institute of Bioscience and Biotechnology, Yuseong-gu, Daejeon, 305-806 Republic of Korea; 5 Severance Integrative Research Institute for Cerebral & Cardiovascular Diseases (SIRIC), Yonsei University College of Medicine, 50 Yonsei-ro, Seodaemun-gu, Seoul, 03722 Republic of Korea; 6 Department of Pathology and Laboratory Medicine, Weill Cornell Medical College, New York, NY USA

## Abstract

In cancer, activation of X-box binding protein (XBP1) has a critical role in tumorigenesis and cancer progression. Transcriptional regulatory mechanism of XBP1 in cancer development has been well known, however, regulation of ubiquitination and degradation of XBP1 has not been elucidated yet. Here we show that Fbw7, a substrate recognition component of the SKP1-Cullin-F-box-type E3 ligase, interacts with XBP1 in a phosphorylation-dependent manner, and facilitates XBP1 ubiquitination and protein degradation. Moreover, Fbw7 inhibits oncogenic pathways including NF-κB, AP1, and Myc induced by XBP1. Interestingly, XBP1 negatively regulates transcription of Fbw7 via a feedback mechanism through NF-κB/E2F-1 axis signaling pathway, suggesting that overexpression of XBP1s may contribute to low level of Fbw7 expression in human cancers. Therefore, a negative feedback loop between Fbw7 and XBP1 contributes to the regulation of tumor development and can be an attractive target for novel therapy in cancers.

## Introduction

X-box binding protein (XBP1) plays a critical role in regulation of endoplasmic reticulum (ER) homeostasis^1^, and is also closely associated with in tumorigenesis and progression of tumor^2^. XBP1s, an active form of XBP1, can be translated from mRNA spliced by the ER stress sensor inositol-requiring enzyme 1α (IRE1a) and regulates multiple target genes such as genes associated with cell proliferation and survival^3^. XBP1s is activated in several cancer cells and activated XBP1s promotes breast cancer progression and metastatic capacity^4^. Overexpression of XBP1s in cancer cells induces drug resistance by regulating cell cycle and apoptosis genes^5^. A recent study has reported that constitutive XBP1s expression promotes tumorigenesis by controlling anti-tumor immunity in dendritic cells^6^. Therefore, proper regulation of XBP1 expression is important for tumor suppression.

However, active form of XBP1, XBP1s, is unstable under common condition. It can be degraded by proteasomes^7^. Therefore, regulation of XBP1s degradation might be able to modulate tumorigenic capacity and tumor progression. Many researches have already demonstrated that transcriptional activation of XBP1 contributes to tumor growth^8,9^. However, the regulation of XBP1s ubiquitination and degradation in cancer cells has not been elucidated yet.

Fbw7 is a substrate recognition component of the Skp1-Cullin-F-box (SCF)-type E3 ligase complex and a well-known tumor suppressor. Fbw7 exerts tumor suppressor function by promoting the ubiquitination and degradation of various oncoproteins including c-Myc, cyclin E, NOTCH-1, and c-Jun^10,11^. Reduced Fbw7 expression and loss-of-function mutations have been demonstrated in various types of human cancer, leading to chromosomal instability and tumorigenesis^12^. Our previous study has shown that Pin1 isomerase can act as an upstream negative regulator of Fbw7 by governing Fbw7 stability and tumor suppressor function^13^. Pin1 interacts with Fbw7 in a phosphorylation-dependent manner. It promotes Fbw7 self-ubiquitination and protein degradation, resulting in the amplification of oncogenic pathways. Thus, Pin1-mediated inhibition of Fbw7 is a key signaling pathway that regulates the stability of various oncoproteins in cancer. Our previous study has also demonstrated that Pin1 regulates XBP1 that has a critical role in cancer signaling^9^. Therefore, we speculate that there are regulatory mechanisms of between XBP1 and Fbw7.

Regulation of transcriptional activation of XBP1 plays a critical role in cancer progression and development. However, at post-translational level, the ubiquitination or degradation of XBP1 has not been well investigated. As XBP1 has also putative Fbw7 consensus sequence, the objective of this study was to determine whether Fbw7 might have a possible role in the regulation of ubiquitination and degradation of XBP1. Furthermore, we investigated the effect of XBP1 on Fbw7 regulatory mechanisms as a feedback loop.

## Results

### Fbw7 interacts with XBP1 in a phosphorylation-dependent manner

**Fig. 1:**
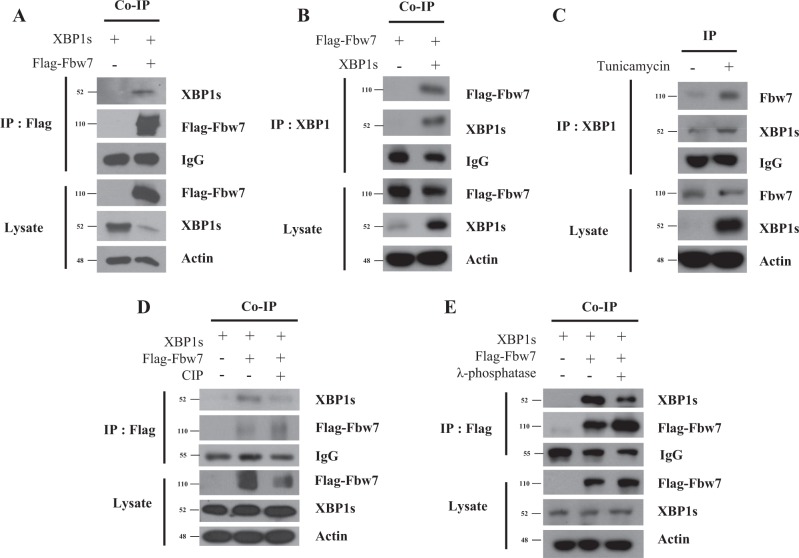
Fbw7 interacts with XBP1s in phosphorylation-dependent manner. **a** Co-immunoprecipitation (Co-IP) of transiently expressed XBP1s and FLAG-Fbw7. HEK293FT cells were co-transfected with XBP1s and FLAG-Fbw7. Immunoprecipitation was detected with anti-FLAG. **b** Co-immunoprecipitation (Co-IP) of transiently expressed XBP1s and FLAG-Fbw7. HEK293FT cells were co-transfected with XBP1s and FLAG-Fbw7. Immunoprecipitation was detected with anti-XBP1. **c** Immunoprecipitation of tunicamycin induced endogenous XBP1s and Fbw7. HEK293FT cells were treated 1 μg/ml tunicamycin 24 h. Immunoprecipitation was detected with anti-XBP1. **d** XBP1s interacts with Fbw7 in a phosphorylation-dependent manner. Transient expressing XBP1s and/or FLAG-Fbw7 lysates were treated with calf intestinal alkaline phosphatase (CIP), and subjected to co-immunoprecipitation. **e** Transient expressing XBP1 and/or FLAG-Fbw7 lysates were treated with λ-phosphatase, and subjected to co-immunoprecipitation. All western blot analyses were carried out with anti-XBP1and anti-FLAG antibodies. Actin was used for an internal control

**Fig. 2:**
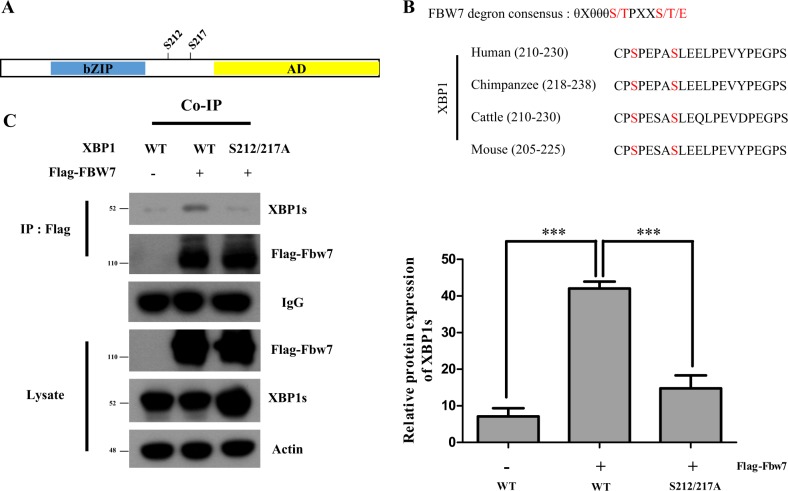
S212/217 site of XBP1 is the direct binding site of Fbw7. **a** Schematic model of the Serine-Proline (SP) sites replaced by alanine. The basic leucine zipper domain (bZIP domain) containing sequence-specific DNA-binding domain and AD sites indicate transcriptional activation domain. **b** Alignment of XBP1s sequence Ser212-Pro site in a variety of species. **c** Transient expressing wild-type (WT) and mutant XBP1s were co-immunoprecipitated with FLAG-Fbw7. HEK293FT cells were co-transfected with XPB1s and FLAG-Fbw7. XBP1s serine 212 and 217 sites were mutated to alanine. Immunoprecipitation was carried out with anti-FLAG. Anti-XBP1 and anti-FLAG were used for immunoblotting. Data are expressed as means ± SD (*n* = 3). ***p < 0.001

It is known that Fbw7, a substrate recognition component of SCF-type E3 ligase complex, is involved in the ubiquitination and degradation of target proteins^13^. XBP1 has putative Fbw7 consensus sequence in amino acid sequences. Thus, we investigated whether XBP1 might be regulated by Fbw7. Notably, we observed an interaction between XBP1 and Fbw7 based on co-immunoprecipitation (Fig. 1a, b). Furthermore, we detected that XBP1 and Fbw7 also interacts in endogenous level (Fig. 1c). This interaction was eliminated by dephosphorylation of XBP1 with calf intestinal alkaline phosphatase (CIP) and λ-phosphatase treatment. (Fig. 1d, e), indicating that Fbw7 could bind to XBP1 in a phosphorylation-dependent manner while XBP1 might be a substrate of the Fbw7 E3 ligase complex. It is known that Fbw7 can bind to specific consensus sequences and that Fbw7 recruitment is promoted by phosphorylation^14^. Thus, we selected a putative Fbw7-binding degron motif on Xbp1 (Fig. 2a) and the putative degron motif on XBP1s at Ser^212^ and Ser^217^ is conserved in various species (Fig. 2b). After substituting those serine sites to alanine, XBP1s wild-type and S212/217 A were co-immunoprecipitated with FLAG-Fbw7. Interestingly, the interaction between Fbw7 and mutant S212/217 A XBP1s was decreased compared with that between Fbw7 and wild-type XBP1s (Fig. 2c). These results suggested that the Ser^212/217^ sites of XBP1s are important for binding to Fbw7.

### Fbw7 promotes ubiquitination and degradation of XBP1

**Fig. 3:**
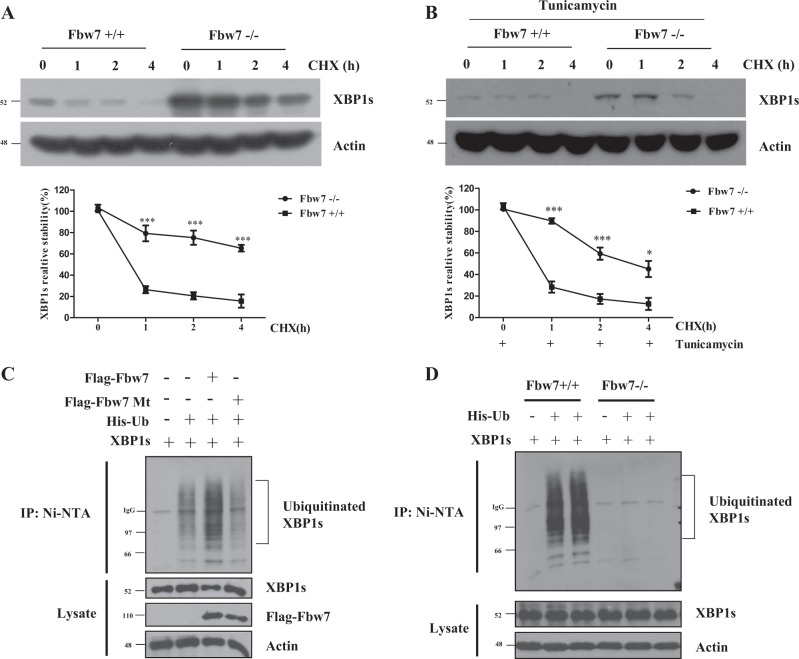
Fbw7 regulates XBP1s ubiquitination. **a** Deficient of Fbw7 leads to increased exogenous XBP1 expression. HCT116 Fbw7−/− cells or HCT116 control cells were transfected with pLenti 6.3 XBP1. Cycloheximide (CHX) at 80 μg/ml was used to treat HCT116 cells at the indicated time points. Western blotting was carried out with the indicated antibodies. **b** Deficient of Fbw7 leads to increased endogenous XBP1 expression. HCT116 Fbw7−/− cells or HCT116 control cells were treated with tunicamycin (TM) which is well-known ER stress inducer. **c** HCT116 Fbw7−/− cells were transfected with Flag-Fbw7 WT (wild type), Mt (mutant), XBP1s, and/or His-tagged ubiquitin (His-Ub) or vector control as indicated. Cells were then lysed in a buffer containing 6 M urea. WCL whole cell lysate. Ubiquitin-conjugated proteins were captured with nickel-agarose beads and subjected to immunoblot analysis with the indicated antibodies. **d** WT of Fbw7−/− HCT116 cells were transfected with His-tagged ubiquitin and/or XBP1s expressing vector. Ubiquitin-conjugated proteins were captured with nickel-agarose beads and subjected to immunoblot analysis with antibodies against XBP1 and actin

A previous study has demonstrated that the expression of XBP1 is increased following treatment with the proteasome inhibitor MG132^9^. Therefore, we hypothesized that XBP1 degradation was mediated by ubiquitination and proteasomes. First, we investigated whether Fbw7 expression affected the protein stability of XBP1s. Cycloheximide (CHX) chase method was used to detect changes in XBP1s stability. In contrast with control cells (FBW7 + / + ), exogenous XBP1s showed higher and longer expression level in Fbw7-deficient cells (FBW7−/−) (Fig. 3a). Furthermore, endogenous XBP1s also indicated higher and longer expression level in Fbw7-deficient cells (FBW7−/−) than control cells (FBW7 + / + ) (Fig. 3b). This suggests that XBP1s is more stable in Fbw7-deficient cells (FBW7−/−), and that Fbw7 can regulate XBP1s expression and destabilize XBP1. Furthermore, we examined XBP1s ubiquitination by Fbw7 expression and detected increased levels of XBP1 ubiquitination in Fbw7-expressing cells compared to that in control vector-expressing cells (Fig. 3c). In contrast, overexpression of inactive Fbw7 mutants did not affect XBP1 ubiquitination. Additionally, ubiquitination of XBP1 was significantly decreased in Fbw7-depleted cells compared to that in Fbw7 wild-type cells (Fig. 3d). These results indicated that Fbw7 can interact with XBP1 in a phosphorylation-dependent manner, and promotes ubiquitination of XBP1, subsequently leading to proteasomal degradation of XBP1s.

### Fbw7 downregulates the function of XBP1 inducing tumorigenesis

**Fig. 4:**
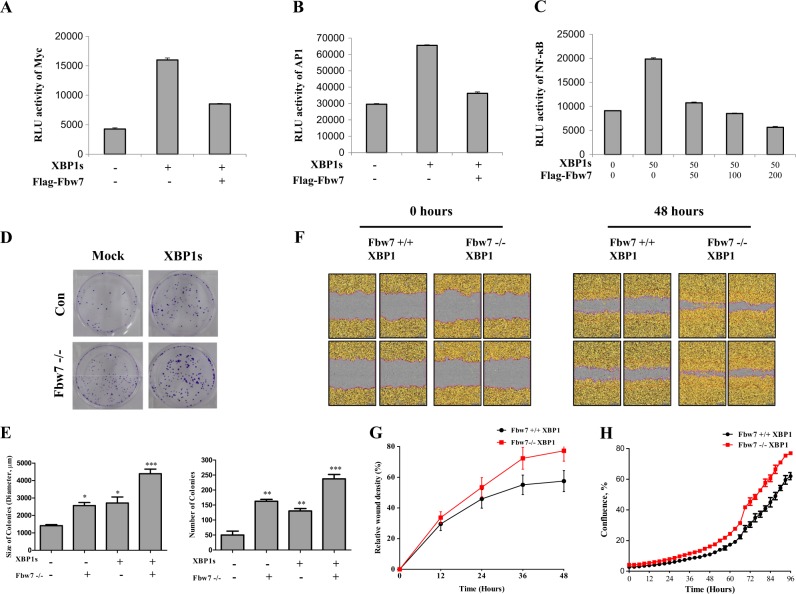
Fbw7 reduces the function of XBP1s activating oncogenic signals. **a** HCT116 cells were co-transfected with the Myc-luc reporter, XBP1s expressing vector, and Flag-Fbw7 expressing vector and then analyzed for luciferase activity at 48 h after transfection. **b** HCT116 cells were co-transfected with the AP1-luc reporter, XBP1s expressing vector, and Flag-Fbw7 expressing vector and then analyzed for luciferase activity after 48 h. **c** HCT116 cells were co-transfected with the NF-κB -luc reporter, XBP1s expressing vector, and Flag-Fbw7 expressing vector and then analyzed for luciferase activity after 48 h. **d** At 10 days after seeding HCT116 control/XBP1 overexpressing/Fbw7 knock down/both XBP1 overexpressing and Fbw7 knock down cells, they were stained with 5% crystal violet. **e** Number and size of colonies from three independent experiments. **f** Scratched HCT116 XBP1-overexpressing cells or HCT116 XBP1-overexpressing cells with Fbw7-null were observed until 48 h using incucyte. **g** Statistical analysis of relative wound density. **h** Proliferation rate of HCT116 XBP1-overexpressing cells or HCT116 XBP1-overexpressing cells with Fbw7-null were detected with Incucyte until 96 h. Data are expressed as means ± SD (*n* = 3). *p < 0.05, **p < 0.01, and ***p < 0.001

Given the important role of Fbw7 in regulating ubiquitination and degradation of XBP1s known to have a critical role in tumorigenesis, we determined whether Fbw7 might affect the cellular function and oncogenic signaling pathways of XBP1s. Our previous study has shown that constitutive activation of XBP1 facilitates several oncogenic signaling pathways including Myc, AP1, and NF-κB activities^9^ Thus, we evaluated the effects of Fbw7 expression on the increased oncogenic signaling pathways by XBP1s using three luciferase reporters (Myc, AP1, and NF-κB). Activities of these three reporters were increased in XBP1s-expressing cells. However, these increased levels of Myc, AP1, and NF-κB were significantly decreased by Fbw7 expression in a dose-dependent manner (Fig. 4a–c). Consistent with the oncogenic function of XBP1, HCT116 cells expressing XBP1s showed a significantly larger focus size as well as increased number of colonies (Fig. 4d, e). Interestingly, depletion of Fbw7 significantly increased the focus number and size, displaying a higher induction effect in XBP1s-overexpressing cells (Fig. 4d, e). Expression of XBP1s in HCT116 cells affected foci formation, similar to that in Fbw7-depleted cells without XBP1s expression (Fig. 4d, e). Migration assay also supported that XBP1s regulation by Fbw7 affects tumorigenic capacity (Fig. 4f, g). In the absence of Fbw7, XBP1s-expressing HCT116 cells significantly promote cancer cell migration compared to HCT116 Fbw7 + / + cells expressing XBP1s (Fig. 4f, g). Cell proliferation assay data also supported that increased XBP1s expression elevates cell proliferation in Fbw7 deficient condition (Fig. 4h). These results together demonstrate that tumorigenic pathways and cellular function of XBP1 are regulated by Fbw7 by reducing the cell transformation capacity of XBP1.

### XBP1s downregulates Fbw7 transcription through NF-κB and E2F-1 pathway as a negative feedback mechanism

**Fig. 5:**
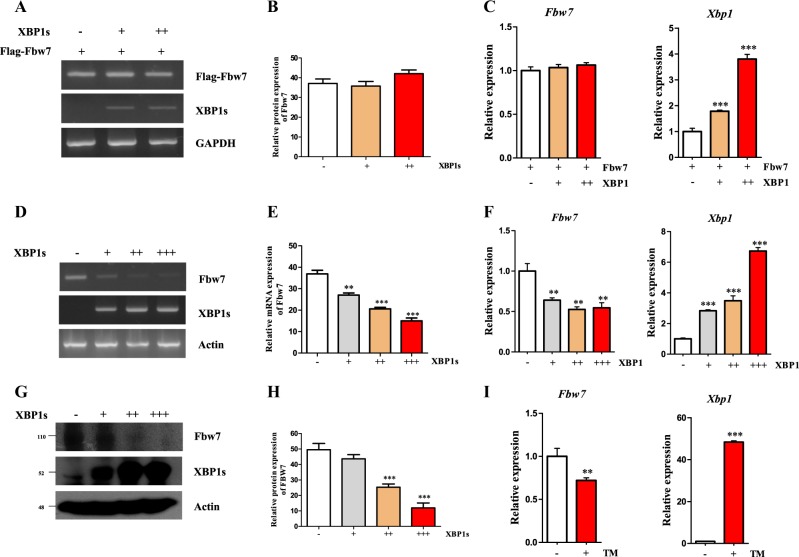
XBP1s downregulates Fbw7 transcription. **a**–**c** HCT116 cells were co-transfected with XBP1s (0, 0.5, and 1 μg) and FLAG-Fbw7 expressing vector. **d**–**f** HCT116 cells were transfected with XBP1s (0, 0.1, 0.5, and 1 μg). Exogenous and endogenous Fbw7 mRNA was detected by reverse transcriptase PCR (RT-PCR) and real-time PCR (qPCR). **g**, **h** HCT116 cells were transfected with XBP1s (0, 0.1, 0.5, and 1 μg). Endogenous Fbw7 protein was detected by western blotting. Western blotting was carried out with indicated antibodies. (I) Effect of tunicamycin (TM) induced endogenous XBP1s on Fbw7 was detected by qPCR. Data are expressed as means ± SD (*n* = 3). *p < 0.05, **p < 0.01, and ***p < 0.001

In addition, we tested the effect of XBP1s on endogenous or exogenous Fbw7 expression. Various concentrations of XBP1s expression plasmid in HCT116 cells were transfected with FLAG-Fbw7 vector to evaluate the expression of exogenous Fbw7 (Fig. 5a–c). Level of exogenous Fbw7 mRNA was not affected by XBP1s. However, the level of endogenous Fbw7 mRNA level was decreased by XBP1s expression in a dose-dependent manner (Fig. 5d–f). Results of western blot indicated that XBP1s expression had a similar effect on Fbw7 expression (Fig. 5g, h). Expression of endogenous XBP1s induced by Tunicamycin also downregulates endogenous Fbw7 mRNA expression (Fig. 5i). These results suggest that XBP1s can downregulate endogenous Fbw7 at the transcriptional level, thereby resulting in the reduction of Fbw7 protein expression.

**Fig. 6:**
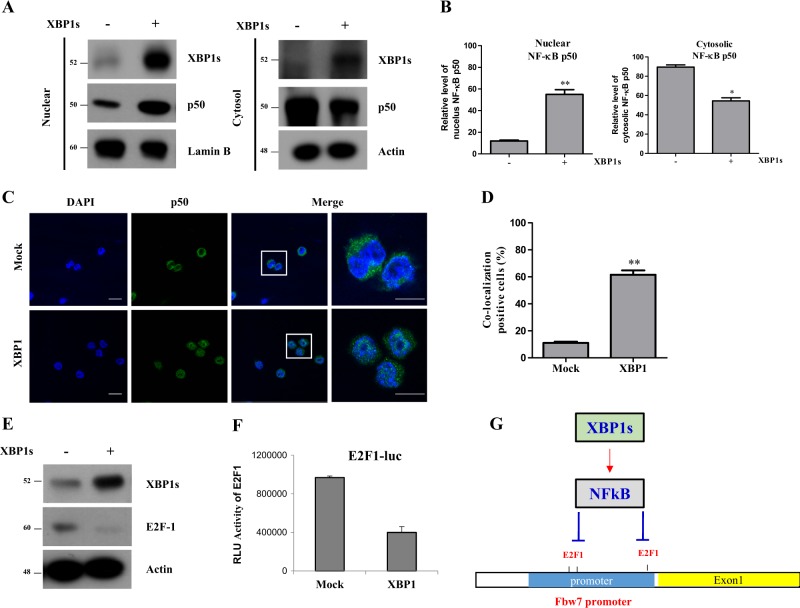
XBP1s inhibits Fbw7 transcription through NF-κB and E2F-1 regulatory pathway. **a** Protein expression levels of NF-κB were detected in HCT116 control and XBP1s overexpressing cells. To detect expression level of NF-κB in cytosol and nuclei, nuclear fraction was prepared. Internal control of the nuclei was Lamin B and that of cytosol was Actin. **b** Quantitative analysis of nucleic and cytosolic NF-κB p50. **c** Nuclei were stained with DAPI. Merged images show the NF-κB and DAPI signals. Scale bar: 10 μm. **d** Quantification of co-localization DAPI and NF-κB p50. **e** Control and stably expressing XBP1s HCT116 cells were used to detect E2F-1 protein expression level. Western blotting was performed with indicated antibodies. **f** HCT116 cells were co-transfected with E2F1-luc reporter and XBP1s expressing vector and then analyzed for luciferase activity after 48 h. **g** Schematic model of regulatory pathway of between XBP1s and Fbw7

We next examined how XBP1s regulated Fbw7 endogenous mRNA level in cells. It has been reported that NF-κB (p50) can downregulate the transcription of Fbw7 by inhibiting E2F-1 transcription factor which activates Fbw7 mRNA transcription^15^. Therefore, we hypothesized that XBP1s can decrease the Fbw7 transcription level by elevating NF-κB activation (nuclear localization of p50). Thus, we tested whether XBP1s affects NF-kB activation (nuclear localization of p50) and E2F-1 expression. Surprisingly, we found that the amount of p50 localized into nucleus from cytosol was increased in XBP1s over-expressing cells (Fig. 6a, b). Immunocytochemistry data also showed increased localization of p50 into nucleus in XBP1s over-expressing cells (Fig. 6c, d). Furthermore, XBP1s expression downregulated E2F-1 expression and E2F-1 luciferase reporter activity upon activation of Fbw7 promoter (Fig. 6e, f). These data suggest that XBP1s can downregulate transcription of Fbw7 through NF-κB/E2F-1 regulatory pathway (Fig. 6g).

## Discussion

Accumulating researches have revealed that ER-stress plays a critical role on growth and survival of tumor cells^16^. XBP1 is one of the most important ER-stress related gene that are involved in tumorigenesis and metastasis of tumor cells^2,17^. Especially, it has been reported that the active form of XBP1 derived from splicing of XBP1 mRNA is associated with cancer development^2^. However, ubiquitination or degradation of XBP1s at the post-translational level has not been elucidated yet. Here we reported that XBP1s can be regulated by ubiquitination and proteasomal degradation via Fbw7 E3 ubiquitin ligase complex. We founded that Fbw7 interacts with XBP1 directly in a phosphorylation-dependent manner and elevates its ubiquitination and subsequent degradation. As a result, Fbw7 inhibited the ability of XBP1s that increases the activities of NF-κB, AP1, and Myc signaling pathways. AP-1 and Myc are known transcription factors and oncoproteins^18,19^. Activation of NF-κB controls stress responses in cancer cells^20^ and cytokine induction in immune cells^12,21^. Therefore, modulation of AP1, Myc, and NF-κB activation through Fbw7-XBP1s axis pathway might affect the progression of cancers and immune response from various stress in humans.

**Fig. 7:**
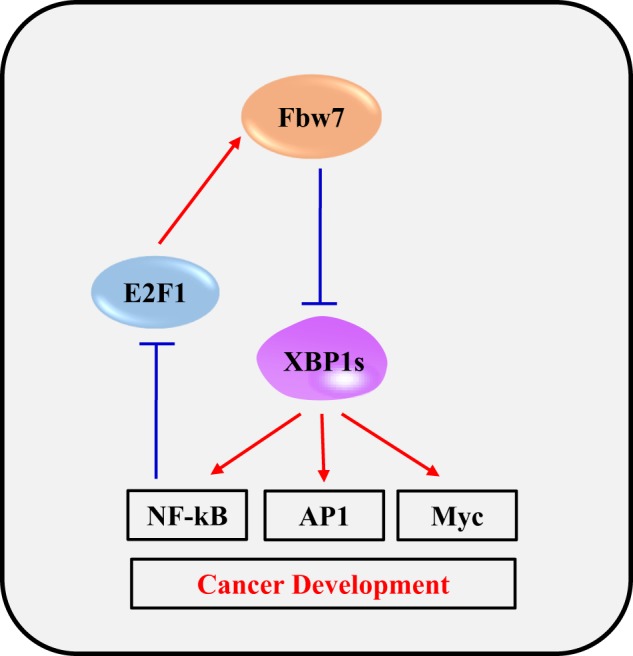
A proposed regulatory mechanism of the Fbw7-XBP1s axis pathway

In a previous study, we have reported that Pin1 interacts with Fbw7 in a phosphorylation-dependent manner and promotes Fbw7 self-ubiquitination and protein degradation, indicating that Fbw7 protein destruction and tumor suppressor function are negatively regulated by Pin1^13^. Our previous study has also demonstrated that there is a negative feedback mechanism between Pin1 and XBP1s through p53^9^. A recent study has suggested that NF-κB p50 downregulates fbw7 mRNA level by inhibiting E2F-1-mediated promoter activation^15^. In the present study, we found that overexpression of XBP1s downregulates transcriptional level of Fbw7 through NF-κB and E2F-1 pathway. Collectively, our results provide a new Pin1-Fbw7-XBP1 signal network including Fbw7-mediated blockage of XBP1s ability to enhance AP1, Myc, and NF-κB activities and a negative feedback mechanism between Fbw7 and XBP1s during tumorigenesis (Fig. 7).

It has been reported that function of Fbw7 serves as a tumor suppressor through negative regulation of oncoproteins in human cancers^22^. Multiple oncoproteins targeted by Fbw7 are undergoing proteasomal degradation through ubiquitination^12^. Therefore, dysregulation of proteolysis by Fbw7 for oncogenic proteins induces promotion of various cancers. Non-functional Fbw7 mutations and low expression of Fbw7 have been shown in many human tumors including bladder, endometrial, and colorectal cancers^23^. Due to the critical role of Fbw7 as a tumor suppressor, investigation of regulatory mechanisms related to Fbw7 is still receiving attention. Here we suggested that XBP1 is one of the regulatory substrate of Fbw7. Besides providing the molecular mechanisms for Fbw7-mediated regulation of XBP1s degradation, we showed that upregulation of Fbw7 decreased the ability of XBP1s to enhance cell transformation whereas depletion of Fbw7 increased XBP1s ability. Interestingly, overexpression of XBP1s downregulated the expression of Fbw7 known to have a critical role in suppressing growth and survival of tumor cells. These results implicated that Fbw7 is one of the upstream regulatory proteins for XBP1s signaling. This further suggest that overexpression of XBP1s may contribute to low level of Fbw7 expression, and low level of Fbw7 or dysfunction of Fbw7 by mutation may contribute to high level of XBP1 expression in human cancers.

In the present study, we demonstrated that a reciprocal regulatory mechanism between XBP1 and Fbw7. Our data provide the molecular mechanism of the Fbw7-XBP1 axis that can be used to propose the new pathway about tumorigenesis. We already presented the regulatory mechanisms between Fbw7 and Pin1 or XBP1 and Pin1. This study suggests a new pathway between XBP1 and Fbw7. Therefore, XBP1, Pin1, and Fbw7 might be closely connected to regulate tumorigenesis. Our findings suggest that the XBP1 and Fbw7 axis might be an attractive target to develop for cancer therapy.

## Materials and methods

### Materials

Cell culture medium and 1% penicillin/streptomycin were purchased from Welgene (Daegu, Korea). Fetal bovine serum (FBS) was purchase from Thermo Fisher Scientific (Waltham, MA, USA) and Atlas biologicals (Fort Collins, CO, USA). CHX, CIP and anti-FLAG-coated magnetic beads were purchased from Sigma (St. Louis, MO, USA). All vectors for cloning and transfection, Lipofectamine 3000, and blasticidin were purchased from Invitrogen (Carlsbad, CA, USA).

### Cell culture and treatment

The HEK (human embryonic kindey)-293FT cell line and HCT116 human colon carcinoma cell line were cultured in DMEM (Dulbecco’s modified Eagle’s medium) containing 10% FBS and 1% penicillin/streptomycin. All cells were incubated at 37 °C in a humidified 5% CO_2_ incubator (SANYO, Osaka, Japan). Cell suspensions were treated with 80 μM CIP for 30 min at 37 °C. Cells were transfected transiently with expression plasmids for the protein stability assay. To block the new protein synthesis, cycloheximide was added. Cells were treated with 1 μg/ml tunicamycin for 24 h at 37 °C.

### Cloning and generation of stable cell line

Coding sequence of XBP1s was amplified by reverse transcription (RT)-PCR with LA Taq polymerase (TaKaRa Bio, Kusatsu, Japan). Amplified XBP1s cDNA was cloned into the pCR8/GW/TOPO vector (Invitrogen) to generate pCR8-XBP1. Subsequently, the pCR8-XBP1 vector was subcloned into the pLenti 6.3/V5-DEST vector to generate 6.3 XBP1. All stable cell lines were generated by transfecting 6.3 XBP1 plasmid into HCT116 and HCT116 Fbw7 knock down cell. XBP-transduced cells were incubated for 72 h and then selected with blasticidin (Sigma) for 1 week. Gene mutation was performed by point mutagenesis service (Bioneer, Daejeon, Korea).

### Immnuoprecipitation and Immnuoblotting

Relevant proteins were transiently expressed in HEK-293FT cells, followed by cell lysis in a buffer as described previously^24,25^. After cell lysis, anti-FLAG-coated magnetic beads (Sigma) or anti-His-tagged agarose beads (Qiagen, Hilden, Germany) were added, followed by further incubation at 4 °C for 2 h. The precipitated proteins were washed in the same lysis buffer and subjected to immunoblotting with anti-FLAG (1:2000; F3165, RRID: AB_259529) (Sigma), anti-XBP1 (1:2000; 619501, RRID: AB_319507) (Biolegend, San Diego, CA, USA) and anti-β-actin (1:4000; #8457, RRID: AB_10950489) (Cell Signaling, Danvers, MA, USA). Cells were also harvested at the indicated time points, and whole-cell-lysates were analyzed by immunoblotting with anti-XBP1 (Biolegend), anti-Fbw7 (1:2000; SC-293423), anti-p50 (NF-κB) (1:2000; SC-8414, RRID: AB_628015), anti-E2F-1 (1:2000; SC-251, RRID: AB_627476) (Santa Cruz Biotechnology, Dallas, TX, USA), anti-β-actin (Cell Signaling), and anti-Lamin B (1:2000; PA 50043) (AB Frontier, Seoul, Korea) antibodies.

### RNA isolation and RT-PCR/qRT-PCR

Total RNA was isolated from HCT116 cells using TRI-solution (Invitrogen). cDNA was synthesized from total RNA with reverse transcription premix (Bioneer). PCR was performed using PCR premix (Bioneer). The following PCR primers were used: 5′-XBP1, 5′-AAACAGAGTAGCAGCGCAGA-3′ and 3′-XBP1, 5′-TCCTTCTGCGTAGACCTCTGGGAG-3′; and 5′ Fbw7, 5′-CCTAAAGAGTTGGCACTCTA-3′ and 3′-Fbw7, 5′-ACTCCACCTGTATGTCCCAC-3′; and 5′-GAPDH, 5′-ACCACAGTCCATGCCATCAC-3′ and 3′-GAPDH, 5′-TCCACCACCCTGTTGCTGTA-3′. In case of Real-time PCR, the synthesized cDNA was amplified with quantitative real-time PCR (StepOnePlus Real-Time PCR system, Thermo) using FastStart SYBR green Master mix (Roche, Basel, Switzerland) and primers. Transcript level of every gene were normalized with GAPDH and ROX dye was used for experiment control. GAPDH was used as reference gene. The results were presented relative to control using the ddCt method. The following qPCR primers were used: 5′-XBP1, 5′-CCCTCCAGAACATCTCCCCAT-3′ and 3′-XBP1, 5′-ACATGACTGGGTCCAAGTTGT-3′; and 5′Fbw7, 5′-GGCCAAAATGATTCCCAGCAA-3′ and 3′Fbw7, 5′-ACTGGAGTTCGTGACACTGTTA-3′.

### Focus formation

Stable HCT116 Con, HCT116 XBP1, HCT116 Fbw7−/−, and HCT116 XBP1 Fbw7−/− cell lines were used for focus formation assay as described previously^26^. In Brief, 500 cells were seeded into 6-well plates. At 10 days after seeding, cells were washed twice times with PBS, fixed with 4% paraformaldehyde (Sigma) and stained with 0.1% Crystal Violet (Sigma) at room temperature for 1 h. Measuring colony sizes and numbers were carried out by using Image J program.

### Scratch wound cell migration assay and cell proliferation

Cells were plated in 96-well ImageLock™ tissue culture plate (Essen BioScience, Ann Arbor, MI, USA) at a density of 4 × 10^4^ viable cells per well and grown in medium. After 24 h, the WoundMaker™ and wounding procedure to create precise and reproducible wounds in all wells of the 96-well ImageLock™ plate. After wounding, aspirate the media from each well and PBS wash each well. After washing, add 100 ml of 1% FBS contained media each well. Remove any bubbles from the assay plate. Place assay plate into the IncuCyte ZOOM^®^ (BioTek, Winooski, VT, USA). Schedule repeat scanning every 2–3 h for 48 h. Cells (500) were seeded in 96-well plates and cultured in DMEM with 10% (v/v) normal FBS for 3 days for measure cell proliferation. The plates were scanned in the IncuCyte imager (Essen Bioscience), and the data were analyzed by the IncuCyte software. Results are representative of three independent experiments.

### Luciferase assay

NF-κB-Luc, Myc-Luc, AP1-Luc, and E2F-Luc reporter plasmids (Promega, Madison, WI, USA) were transfected into cells using Lipofectamine 3000 for the luciferase assay. DNA sample was mixed with 100 ng of CMV-Ren plasmid (Promega) as an internal control and co-trnasfected into cells in 6-well plates. Luciferase assay were performed at 48 h after transfection using Luciferase Assay Reagent kit (Promega) and a Synergy NEO (BioTek, Winooski, VT, USA). Values obtained were normalized to Renilla luciferase activity.

### Nuclear/cytosol isolation and immnuocytochemistry

Nuclear and cytosol fractions were prepared using a Nuclear and Cytoplasmic Isolation kit (Thermo Scientific). All cells used for immunocytochemistry were fixed with 4% paraformaldehyde (Sigma) and permeabilized with 0.25% Trition X-100 in phosphate-buffered saline containing 1% bovine serum albumin. Fixed cells were incubated with an anti p50 (NF-κB) (1:500; SC-8414, RRID: AB_628015) antibody (Santa Cruz Biotechnology) at 4 °C overnight. After that, cells were incubated with Alexa 488 goat anti-mouse secondary antibody (Thermo Scientific) at 4 °C overnight. Images were obtained using an LSM-710 confocal microscope (Carl Zeiss, Oberkochen, Germany).

### Statistical analysis

Prism Software (GraphPad Prism version 5.0, La Jolla, CA, USA) was used for all statistical analyses. Data are presented as mean ± SD of at least three independent experiments (*n* ≥ 3). Dunnett’s multiple comparison test was performed for comparisons among groups. A *p-*value of <0.05 was considered statistically significant. It is indicated by an asterisk in graphs. *P-*values <0.01 and 0.001 are indicated by two and three asterisks, respectively.

## Supplementary information

Supplementary figure 1
