# Supplementary material for: A negative feedback loop between XBP1 and Fbw7 regulates cancer development
Source: Oncogenesis. 2019 Feb 19;8(3):12. doi: 10.1038/s41389-019-0124-4 (PMC6381103; doi:10.1038/s41389-019-0124-4)
Supplement: Supplementary file 1 — Supplementary figure 1 [file 41389_2019_124_MOESM1_ESM.pptx]

## Slide 1
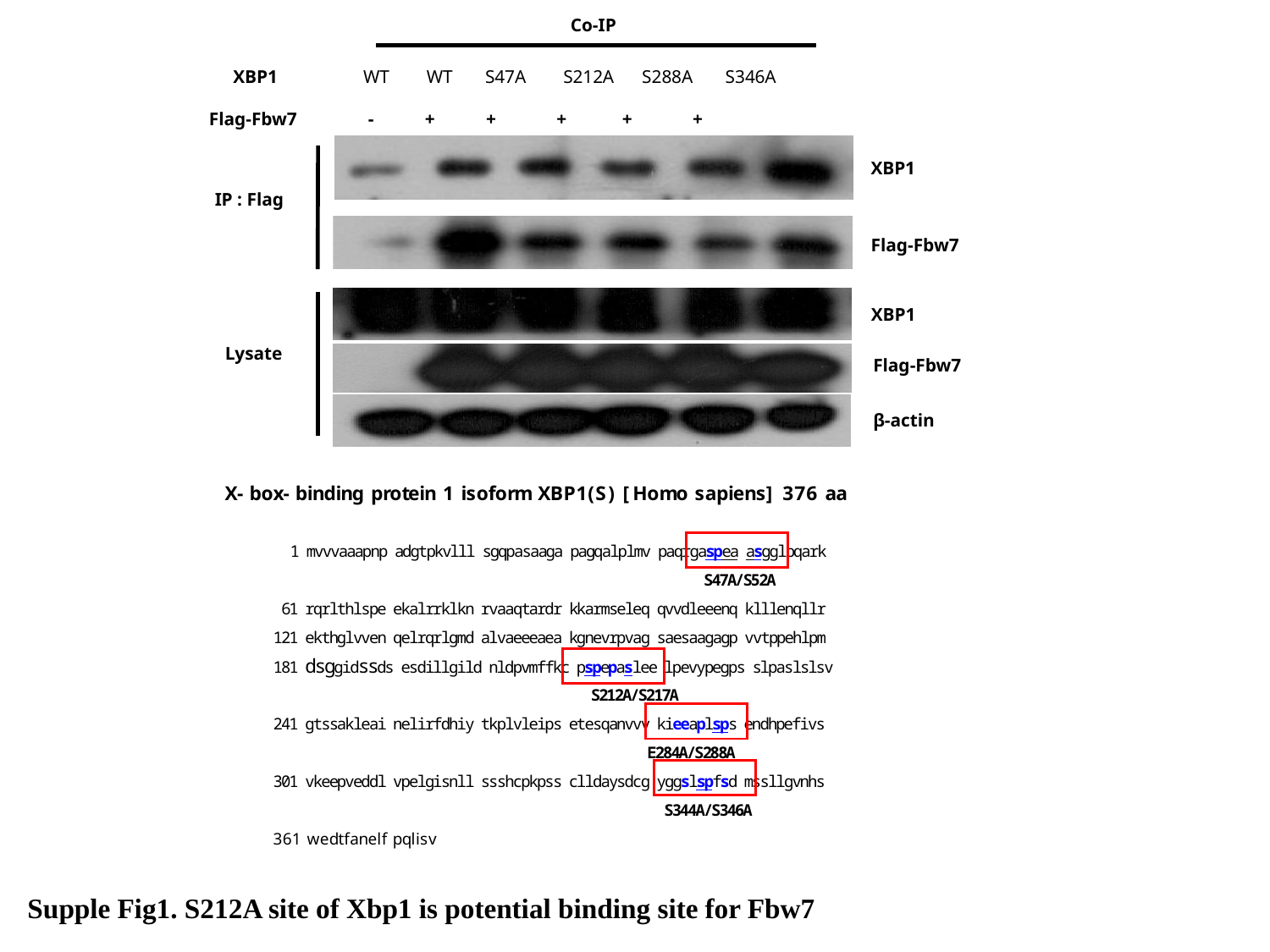

Co-IP
 WT WT S47A S212A S288A S346A
 - + + + + +
 XBP1
Flag-Fbw7
XBP1
IP : Flag
Flag-Fbw7
XBP1
Lysate
Flag-Fbw7
β-actin
Supple Fig1. S212A site of Xbp1 is potential binding site for Fbw7
